# Recent Advances in Diagnostic Approaches for Mucormycosis

**DOI:** 10.3390/jof10100727

**Published:** 2024-10-19

**Authors:** Jawad Safiia, Marco Aurelio Díaz, Hassan Alshaker, Christine J. Atallah, Paul Sakr, Dimitrios G. Moshovitis, Ahmad Nawlo, Andres E. Franceschi, Alexis Liakos, Sophia Koo

**Affiliations:** 1 Brigham and Women’s Hospital, Boston, MA 02115, USA; jawad.safiia@hotmail.com (J.S.); mdiaz33@bwh.harvard.edu (M.A.D.); halshaker@bwh.harvard.edu (H.A.); catallah@bwh.harvard.edu (C.J.A.); psakr@bwh.harvard.edu (P.S.); dmoshovitis@bwh.harvard.edu (D.G.M.); anawlo@bwh.harvard.edu (A.N.); afranceschicoll@gmail.com (A.E.F.); aliakos@bwh.harvard.edu (A.L.); 2 Dana-Farber Cancer Institute, Boston, MA 02215, USA; 3 Harvard Medical School, Boston, MA 02115, USA

**Keywords:** mucormycosis, Mucorales, metabolomics, volatile organic compounds, medical mycology, fungal biomarkers

## Abstract

Mucormycosis, an invasive fungal infection caused by members of the order Mucorales, often progresses fulminantly if not recognized in a timely manner. This comprehensive review discusses the latest developments in diagnostic approaches for mucormycosis, from traditional histopathology and culture-based methods to advanced and emerging techniques such as molecular assays, imaging, serology, and metabolomics. We discuss challenges in the diagnosis of mucormycosis and emphasize the importance of rapid and accurate identification of this life-threatening infection.

## 1. Introduction

Mucormycosis is a serious opportunistic infection caused by ubiquitous molds belonging to the order Mucorales, of which the most commonly isolated genera are *Rhizopus*, *Mucor*, *Cunninghamella*, and *Lichtheimia* [1,2]. Infection is generally acquired through inhalation of sporangiospores, with rhino-orbito-cerebral mucormycosis (ROCM) and pulmonary mucormycosis being the most common clinical syndromes [2]. Risk factors frequently associated with mucormycosis include hematological malignancy, diabetes mellitus (especially in patients with ketoacidosis), glucocorticoid exposure, and neutropenia [2].

Mucorales are the second most common cause of filamentous fungal infections after *Aspergillus* spp., although the absolute cumulative incidence of mucormycosis is relatively low [3,4,5]. However, an ever-expanding population of diabetic and immunocompromised patients is driving the increase in cases seen in recent years, which warrants considering mucormycosis an emerging infection [6]. In particular, the COVID-19 pandemic has driven a dramatic increase in cases, especially in India [7]. COVID-19-associated mucormycosis confers a mortality risk as high as 38.9% [8].

Despite being an infection seldom encountered by many clinicians, mucormycosis is of high significance due to its disproportionately high rates of fatal outcomes. The overall survival rate of ROCM is 59.5%, a figure which has remained stable over the last two decades despite advances in novel broad-spectrum mold-active triazoles with activity against Mucorales, such as posaconazole and isavuconazole [9]. Pooled mortality for pulmonary mucormycosis also remains extremely high at 57.1% [10].

Mucorales most often cause rapidly progressive invasive infections, and the frequency of fatal outcomes in mucormycosis is partly explained by their distinctive propensity for angioinvasion, resulting in vessel thrombosis and ischemic tissue necrosis. After sporangiospore germination into hyphae, specific recognition and invasion of endothelial cells by the fungus is mediated by the interaction between fungal ligands belonging to the spore coating (CotH) protein family and the host receptor glucose-regulator protein 78 (GRP78), both of which are expressed to a greater extent in experimental models of diabetic ketoacidosis [11,12,13,14]. Delays in surgical debridement and appropriate antifungal therapy are associated with increased mortality [9,10,15]. However, diagnosis of mucormycosis remains a tremendous challenge, as reliable tests allowing for rapid and accurate diagnosis are limited and mostly in an early development stage. Current diagnostic strategies center around invasive or slower techniques such as histopathology, fungal cultures, or polymerase chain reaction (PCR). Novel, culture-independent diagnostics that do not rely on these techniques would allow for shorter turnaround times (TAT) and potentially improved survival for patients. Factors that contribute to the difficulty in diagnosing these infections include the absence of consensus on diagnostic pathways, the limited sensitivity of conventional diagnostic methods, the need for invasive procedures for definitive diagnosis in many cases, and the fact that many promising diagnostics lack methodological standardization and validation.

## 2. Imaging

**Figure 1:**
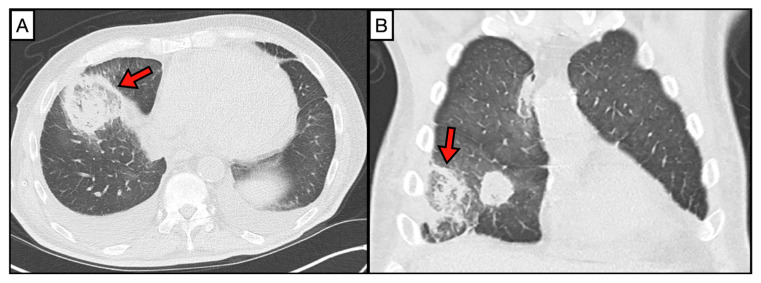
Reverse halo sign (red arrow) in (**A**) axial and (**B**) coronal chest CT images from a patient with pulmonary mucormycosis caused by *Rhizopus arrhizus*.

Using chest roentgenograms of six patients between 1968 and 1972, Bartum et al. identified nonspecific consolidations with cavitations, infarcts, and infiltrates in patients whose autopsies ultimately revealed mucormycosis post-mortem. Since these findings can also occur in patients with *Candida* or *Aspergillus* infections, they determined that mucormycosis cannot be differentiated from candidiasis or aspergillosis using X-ray imaging [16]. Using CT imaging, subsequent studies found radiographic features that generally differentiate mucormycosis from invasive pulmonary aspergillosis (IPA). For example, 64% of immunocompromised patients with pulmonary mucormycosis (PM) were found to have a higher number of lung nodules (10 or more) and 55% a higher number of micronodules (<1 cm) when compared to immunocompromised patients with IPA. Additionally, pleural effusions were observed in 63% of patients with PM but only in 33% of patients with IPA [17]. The ‘halo sign’ was present in 25% of patients with PM and 21% of those with IPA. As with late IPA, PM patients can also have the ‘air crescent’ sign on CT and X-ray imaging, although rarely [18]. The ‘reverse halo’ sign (RHS), in which a rim of dense consolidation surrounds an infarct, is more prevalent in patients with mucormycosis compared to patients with IPA (Figure 1). The RHS was seen in 19% of patients with PM in one retrospective study of eight patients who predominantly had underlying leukemia, while it was observed in <1% of patients with IPA and in no patients with pulmonary fusariosis [19]. Another study found that 94% of patients with leukemia diagnosed with PM, all in the context of neutropenia, had the RHS [20]. This study also found micronodules in 64% of patients with PM about 1–2 weeks after the first CT scan following diagnosis, but not within the first week [20], corroborating prior studies. Combined, these studies suggest that CT findings, such as the RHS, presence of micronodules, a large number of nodules, and pleural effusions, are more commonly found in patients with PM than IPA or other invasive mycoses.

Beyond PM, CT imaging can be a useful adjunct in the diagnosis of rhino-orbital-cerebral mucormycosis (ROCM). A retrospective study reviewing CT images in immunocompromised patients with invasive fungal sinusitis, including cases caused by *Aspergillus* and Mucorales, determined that 91% of patients had thickening of the nasal cavity mucosa, turbinates, nasal septum, and nasal floor, and thickening or opacification of the sinus mucoperiosteum; only 20% of patients with non-fungal etiologies of their sinusitis had these findings [21]. In 8% of IFS cases, bony erosion was also present, which was not a finding in patients with non-fungal sinusitis in this study [21]. Significant involvement of the nasal floor and lateral wall in the premaxillary or retromaxillary areas, hard palate, pterygomaxillary fossa and pterygopalatine bone, medial wall of the orbit, and bilateral orbital bone were more specific imaging findings of ROCM compared to patients with non-Mucorales etiologies of their sinusitis [22].

In addition to CT imaging, a recent study suggested that MRI is also a potentially useful modality for identifying signs of ROCM. As many as 75% of patients with suspected ROCM in the setting of COVID-19 have MRI findings such as mucosal thickening, sinus opacification, and air-fluid levels, with 41% having orbital, periorbital, and maxillofacial involvement, 33% bony involvement, 15% brain involvement, and 11% optic nerve involvement [23]. Patil et al. proposed an MRI-based scoring system to grade ROCM, allocating points for bone marrow edema, sinus involvement, intracranial extension, optic nerve involvement, and orbital, periorbital, or maxillofacial soft tissue extension to classify cases as mild, moderate, or severe [23].

Although PET imaging is not a first-line modality for diagnosing mucormycosis, [^18^F]FDG uptake is increased in the lungs of patients with probable and definite PM and with comparable sensitivity to CT and MRI for diagnosing PM, among other invasive fungal infections [24]. [^18^F]FDG uptake can be increased in bone and soft tissues, including the sinuses and orbital regions, in patients with definitive ROCM, and it can also be useful in monitoring the therapeutic response to antifungal therapy [25,26]. In gastrointestinal mucormycosis, [^18^F]FDG-PET can also show [^18^F]FDG uptake at the site of infection. However, it is difficult to distinguish from other metabolically active processes such as malignancy other than relatively greater uptake of [^18^F]FDG in the periphery of the lesion compared to the center [27,28]. Similarly, intense peripheral FDG uptake compared to the center of mass-like lesions is also seen in PM [29].

## 3. Diagnostic Approaches Based on Morphology

As with all invasive fungal infections, the “gold standard” for diagnosing mucormycosis definitively involves a combination of histopathology and culture. Histopathology and culture serve as the benchmark against which novel diagnostics are compared in terms of turnaround time (TAT), cost-effectiveness, sensitivity, and specificity. A positive culture obtained from a normally sterile site or histopathology demonstrating hyphal forms with morphology consistent with mucormycosis are required to diagnose mucormycosis definitively, but many patients with suspected mucormycosis are unable to tolerate invasive biopsy procedures, and culture yield from biopsies can be unpredictable [30]. If feasible, tissue biopsies should be obtained during the diagnostic workup of patients with suspected mucormycosis, given the rapid progression of this infection if not diagnosed in a timely manner [31].

### 3.1. Direct Microscopy of Clinical Specimens

Septate versus coenocytic (i.e., non-septate) hyphal morphologies can be differentiated using direct microscopic examination of wet mount preparations or fixed stained smears of clinical material from tissue biopsy specimens. Direct microscopy findings of broad, non-septate hyphae should be considered meaningful coming from a normally sterile site in a patient with a compatible clinical syndrome, even if fungal cultures are ultimately sterile [32]. Discerning whether a positive finding represents infection or merely colonization can be challenging, however, when samples are taken from a non-sterile body site.

### 3.2. Histopathology

**Figure 2:**
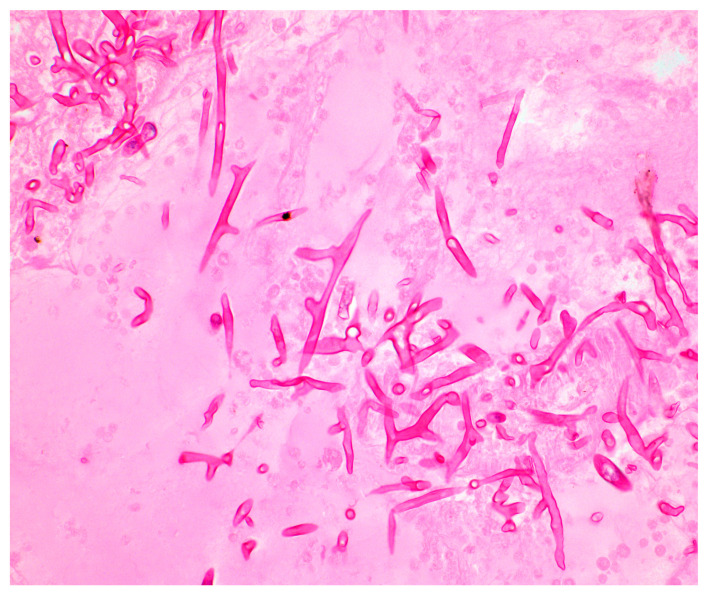
Hematoxylin–eosin (H&E) stained slide demonstrating the broad, ribbon-like pauci-septate, 90-degree angle branching hyphae of *Rhizopus* in a patient with disseminated infection in the brain tissue. Image courtesy of Dr. Isaac Solomon.

**Figure 3:**
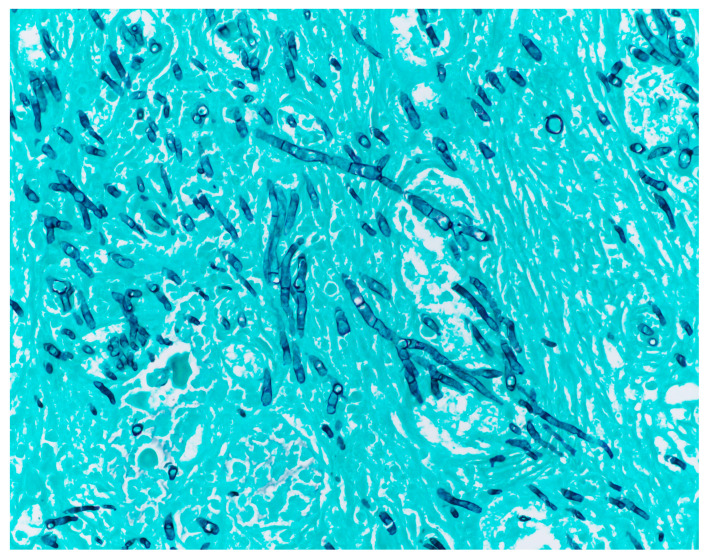
Grocott–Gömöri’s methenamine silver (GMS) stained slide showing more narrow, septate, acute-angle branching hyphae of *Aspergillus fumigatus* in a patient with invasive aspergillosis.

In hematoxylin–eosin (H&E)-stained slides, Mucorales classically appear as nonpigmented, broad (5–20 μm), ribbon-like, thin-walled, coenocytic or pauci-septate, 90-degree branching hyphae invading blood vessels [33,34] (Figure 2). Interstitial pressure and drying of the tissues on fixation tend to distort fungal morphology, however, and Mucorales hyphae can sometimes falsely appear to have acute-angle branching and pseudoseptations. This can result in interpretive errors which lead to mistaking mucormycosis for aspergillosis (Figure 3), and it is important that pathologists experienced in reviewing hyphal morphology examine these biopsy samples [30,35]. Additionally, Mucorales hyphae are labile and prone to fragmentation during tissue homogenization for histopathology, which also contributes to reduced sensitivity [36]. Mucorales do not readily take up Grocott–Gomori’s methenamine silver stain (GMS) compared to other fungi, and although various modifications to the technique have been proposed to increase sensitivity, they are not standardized [37,38]. TAT, from biopsy sample collection to pathology results, can range from several hours to several days; an additional method to improve TAT in this context is the examination of intraoperative frozen sections, particularly for ROCM. A recent meta-analysis of data from 458 patients with acute invasive fungal rhinosinusitis who underwent intraoperative sampling for frozen section reported a sensitivity of 75.4% and specificity of 98.6% for identifying Mucorales, and a positive predictive value of 98.2% for all etiologies of acute invasive fungal rhinosinusitis [39]. This supports the use of intraoperative frozen sections as an adjunctive diagnostic method to guide empiric antifungal therapy directed against Mucorales before obtaining definitive pathology and culture results. One of the shortcomings of histopathologic examination is that it does not allow for species-level identification, although tissue with hyphae consistent with Mucorales can be sent for molecular sequencing (as discussed in Section 5.1 below).

### 3.3. Immunohistochemistry

For cases with indeterminate fungal hyphal morphology on histopathology, various immunohistochemistry (IHC) assays have been investigated to increase diagnostic sensitivity in those patients with a tissue biopsy showing hyphal forms. An IHC assay targeting a *Rhizopus arrhizus* antigen demonstrated sensitivity of 100% and specificity of 100% in one study, with a follow-up study showing 97% concordance between IHC and positive cultures in 60 out of 62 patients [40,41].

Other methods include fluorescence in situ hybridization (FISH) targeting the rRNA genes of Mucorales. This method was found to have a sensitivity of 79% for differentiating mucormycosis from other fungal species [42]. Another study compared the sensitivity of IHC to FISH, with 7 of 17 samples (41%) positive by IHC compared to 12 (71%) samples positive by FISH [43].

### 3.4. Fungal Cultures

Mucorales generally grow on conventional Sabouraud dextrose agar in 3–7 days [44,45]. Despite being angioinvasive fungi, blood cultures almost never grow Mucorales and are of very limited utility in the diagnosis of mucormycosis [32,45]. The ideal sample for culture is a biopsy specimen from a normally sterile anatomic site, processed with tissue slicing instead of grinding, without prolonged refrigeration and incubated on Sabouraud dextrose agar plates at 30 °C and 37 °C [31]. These conditions are often not all met simultaneously in biopsy samples, with the proportion of patients with mucormycosis whose biopsies yield Mucorales in culture varying widely in the literature. In a recent meta-analysis of 851 patients with proven or probable mucormycosis, 79% of biopsy samples had positive culture results [2]. Therefore, a negative culture from a biopsy specimen does not rule out mucormycosis. Growth of Mucorales in culture allows for species-level identification of Mucorales and antifungal susceptibility testing. This advantage, shared with molecular sequencing techniques, is critical given how the newer mold-active triazoles posaconazole and isavuconazole appear to have species-specific differential in vitro activity against the Mucorales [46]. For instance, isavuconazole has been reported to exhibit favorable in vitro activity against *Rhizopus arrhizus* but reduced in vitro potency against *Mucor circinelloides* [47]. Despite often long TAT and unpredictable yield, fungal culture remains an important part of the current gold standard for diagnosing mucormycosis.

## 4. Serology and Biomarkers

The fungal cell wall biomarkers (1→3)-β-d-glucan and galactomannan are not present in the cell wall of Mucorales, so they cannot be used as a diagnostic adjunct in mucormycosis [48,49]. There are currently no commercially available antigen tests for diagnosing mucormycosis, with the methods described below being used in research only at this time.

An older ELISA-based serum assay targeting *Rhizopus arrhizus* and *Rhizomucor pusillus* antigens was described to have a sensitivity of 81% and specificity of 94% but with some molecular cross-linking with *Aspergillus* and *Candida* [50]. A more recent lateral flow immunoassay (LFIA) targeting α-1,6-mannans common to *Mucor* and *Rhizopus* species potentially has higher sensitivity for Mucorales compared to *Aspergillus*, *Candida*, and *Fusarium* in an experimental setting [51]. Another LFIA assay targeting a fucomannan antigen on the Mucorales cell wall has been shown to have superior diagnostic performance to traditional ELISA in the serum, bronchoalveolar lavage (BAL) fluid, and urine in murine models [6]. A lateral flow device is also being investigated for use in patients with poorly controlled diabetes and on high-dose corticosteroids, targeting the 15 kDa extracellular polysaccharide (EPS) antigen secreted by *Rhizopus oryzae* and *Rhizopus delemar* during their growth phase [52]. These lateral flow techniques warrant further investigation and have the potential to allow more rapid TAT in the diagnosis of mucormycosis than is currently possible, especially in lower-resourced environments.

## 5. Molecular Assays

PCR testing for early detection of Mucorales in high-risk patients has been a thriving area of investigation, with studies of multiple assays performed on various bodily fluids and tissue (Table 1).

### 5.1. Molecular Assays in Tissue Samples

Histopathological diagnosis of mucormycosis is traditionally based on assessment of hyphal morphology in tissue biopsies, which requires a high level of pathologist expertise and is often subject to hyphal fragmentation, with suboptimal sensitivity [34,53,54]. PCR can be used for direct identification of Mucorales from tissue specimens, mitigating some of these issues [55].

A panfungal PCR has been used to amplify the ITS1 region of the rDNA gene, followed by DNA sequencing, with >97% sensitivity in fresh tissue and 68% in formalin-fixed paraffin-embedded (FFPE) specimens [56,57]. Panfungal PCR assays can detect any fungal DNA sequence, even from uncultured or rare fungi [58]. However, limitations of panfungal PCR include the need for sequencing of amplicons for species determination, which prolongs the TAT, and the potential for false positive results due to fungal contamination or colonization of non-sterile sites such as the airways or sinuses [56,59,60,61,62,63,64]. Panfungal PCR assays of tissue samples have widely varying estimates of sensitivity and specificity for mucormycosis, depending on the type of tissue sample and primers used. One study reported 58%, 34%, and 100% sensitivity for different rDNA targets using FFPE samples. Another study reported 98% sensitivity for 18S rRNA sequencing compared to 87% for ITS sequencing in 233 clinical samples. Another study reported 90% sensitivity for mucormycosis using primers adapted for quantitative PCR [62,65]. An in-house panfungal PCR was tested on 105 clinical samples from patients with suspected invasive fungal infection, with a sensitivity of 90.4% and a specificity of 79.2% for mucormycosis [61,65].

Multiple other PCR techniques, including semi-nested PCR using Mucorales-specific 18S rDNA primers, real-time PCR with high-resolution melt analysis, multiplex qPCR targeting ITS1/ITS2, cytochrome b gene-specific qPCR, 28S rDNA-specific qPCR, qPCR assays targeting 18S and 28S regions, and PCR coupled to ESI-MS have been assessed in the diagnosis of mucormycosis, with the choice of target and tissue biopsy size affecting results [65,66].

The diagnosis of mucormycosis using PCR of tissue samples faces challenges, including a lack of standardization, DNA degradation in formalin-fixed paraffin-embedded (FFPE) tissues significantly impacting sensitivity compared to the use of fresh tissue samples, and the risk of contamination due to the ubiquitousness of Mucorales in the environment [62].

### 5.2. Molecular Diagnosis in Serum/Blood Samples

Mucormycosis is characterized by aggressive angioinvasion and early hematogenous spread, leading to detectable DNA levels in the blood of some patients [67]. Detection of Mucorales DNA in serum samples with real-time qPCR can facilitate the noninvasive and species-specific diagnosis of mucormycosis. Several studies have examined the diagnostic utility of screening high-risk patients with hematological malignancy and severe burns with these assays [68,69]. A combination of real-time qPCR assays using 18S rRNA primers from the most common pathogenic genera (*Mucor/Rhizopus*, *Lichtheimia*, *Rhizomucor*, *Cunninghamella*) has been described to have a sensitivity of 81% to 92% for mucormycosis [70].

In a retrospective study of archived serum samples from 44 patients who were ultimately diagnosed with mucormycosis, a multiplex serum qPCR assay targeting *Mucor/Rhizopus*, *Rhizomucor*, and *Lichtheimia* was first positive a median of 9 days before radiographic diagnosis, with high concordance between qPCR and tissue-based identification [71]. Patients with a primary lesion larger than 3 cm in size had a higher rate of serum qPCR positivity [72]. Another study using a different PCR assay on blood samples described a sensitivity of 79.3%, specificity of 86.4%, positive predictive value of 65.7%, and negative predictive value of 92.7% [73]. The MODIMUCOR multicenter prospective study in patients with suspected invasive fungal disease found that the multiplex qPCR assay targeting *Mucor*/*Rhizopus*, *Rhizomucor*, and *Lichtheimia* sequences had a sensitivity of 85.2% and specificity of 89.8% in serum. Mucorales qPCR was positive in serum 4 days before mycological or histopathological examination and 1 day before first imaging findings potentially suggestive of invasive mucormycosis. Despite antifungal therapy, patients with persistently positive qPCR had a 100% mortality rate at 6 months, whereas those with negative qPCR within 7 days after initiation of liposomal amphotericin B had a significantly better clinical outcome [74].

### 5.3. Molecular Diagnosis in BAL and Urine Fluids

PCR in combination with high-resolution melt analysis (PCR/HRMA) has been used to detect Mucorales in bronchoalveolar lavage (BAL) samples, with a reported sensitivity of 100% and specificity of 93% in one study [75]. Another study described the use of quantitative PCR (qPCR) on BAL fluid, with a sensitivity of 100% and specificity of 97% for invasive mucormycosis [76].

The diagnostic performance of PCR assays in pulmonary mucormycosis (PM) is influenced by host factors such as neutropenia. The sensitivity of serum qPCR is higher in neutropenic patients, while BAL qPCR may be more informative in non-neutropenic patients [72].

Spore coat protein homolog *CotH* genes in Mucorales enable host cell invasion through GRP78 binding [12]. *CotH* may be a unique diagnostic target in mucormycosis. In mice infected with common Mucorales species, including *Rhizopus delemar*, *Lichtheimia corymbifera*, *Rhizopus oryzae*, *Mucor circinelloides*, and *Cunninghamella bertholletiae*, *CotH* was detectable in plasma, urine, and BAL samples within 24 h of infection, with a diagnostic sensitivity of 90% and specificity of 100%, with urine samples being particularly reliable [63].

### 5.4. Next-Generation Sequencing (NGS)

Next-generation sequencing (NGS) can identify microbial genome sequences [77]. It has been used as a method to identify a broad range of pathogens using DNA or RNA extracted from clinical samples, including blood, BAL fluid, and urine [78]. NGS, in theory, can yield diagnostic information in 24–48 h, depending on the pipeline [15,79]. Some studies have described ex-RNA extracted from extracellular vesicles (EV) to be potential biomarkers in patients with invasive mucormycosis caused by *R. delemar* [80]. In pulmonary infections, including pulmonary fungal infections, NGS may be more sensitive in BAL fluid than in blood samples (81.3% vs. 25.0%, *p* = 0.003) [81]. Limitations of NGS include the high cost of these assays and the need for specialized reference laboratories [82]. Moreover, false positive results may occur due to contamination and the presence of non-disease-causing pathogens, making a clinical assessment of the significance of NGS results in the context of the patient’s risk factors and clinical syndrome essential [83].

**Table 1:** Comparison of the sensitivity and specificity between various serological tests, molecular assays.

Assay Type	Sample Type	Sensitivity (%)	Specificity (%)	Comments
**Serology**				
ELISA (*Rhizopus arrhizus* and *Rhizomucor pusillus*) [50]	Serum	70–81	90–94	Some cross-reactivity with *Aspergillus* and *Candida*.
LFIA (α-1,6-mannans) [51]	Serum	70–90 (experimental)	N/A	Higher sensitivity for Mucorales compared to *Aspergillus*, *Candida*, and *Fusarium* (experimental).
LFIA (Fucomannan antigen) [6]	Serum, BAL, Urine	80–95 (experimental)	N/A	Superior diagnostic performance in murine models.
LFIA (15 kDa EPS antigen) [52]	Serum	N/A	N/A	Under investigation for patients with poorly controlled diabetes and on high-dose corticosteroids.
**Molecular Diagnosis**				
Panfungal PCR (ITS1 region) [56,57]	Fresh tissue	85–97	N/A	High sensitivity in fresh tissue.
Panfungal PCR (ITS1 region) [56,57]	FFPE tissue	40–68	N/A	Lower sensitivity in formalin-fixed paraffin-embedded specimens.
Panfungal PCR (18S rRNA sequencing) [62,65]	Tissue	87–98	N/A	High sensitivity for 18S rRNA sequencing in clinical samples.
In-house panfungal PCR [61,65]	Tissue	58–90	70–80	Tested on 105 clinical samples for invasive fungal infection.
Multiplex qPCR (*Mucor*/*Rhizopus*, *Lichtheimia*, *Rhizomucor*, *Cunninghamella*) [70]	Serum	81–92	N/A	High-risk patients with hematological malignancy and severe burns.
Multiplex qPCR (MODIMUCOR study) [74]	Serum	85	89–90	Multicenter prospective study qPCR positive before mycological or histopathological examination.
qPCR (various assays) [73]	Serum	79–85	85–90	Different PCR assays on blood samples, high negative predictive value.
PCR/HRMA [75]	BAL	90–100	90–93	High-resolution melt analysis.
qPCR [76]	BAL	90–100	95–97	High sensitivity and specificity for invasive mucormycosis.
*CotH* genes (qPCR) [63]	Urine, BAL, Plasma	85–90	100	*CotH* gene target detectable within 24 h of infection in murine models, urine samples particularly reliable.
NGS [77,78,81]	Blood, BAL, Urine	81.3% (BAL), 25% (blood)	N/A	More sensitive in BAL fluid than in blood for pulmonary mucormycosis.

## 6. Metabolomics

Microbes, including molds, emit a variety of distinctive volatile organic compounds (VOCs) in the course of their metabolism, with some synthetic pathways and metabolic products that are not present in humans, with many such VOCs observed in Mucorales. These microbial VOCs can be used as markers for the noninvasive, breath-based diagnosis of mucormycosis and other invasive fungal infections such as aspergillosis [84,85]. In a prior investigation of VOCs in invasive mucormycosis, our laboratory examined breath-volatile metabolite profiles in a murine experimental model. Our focus was on three of the most common Mucorales species that cause human IM: *Rhizopus arrhizus* var. *arrhizus*, *R. arrhizus* var. *delemar*, and *R. microsporus*. We used a murine ventilator to collect breath samples and thermal desorption gas chromatography/tandem mass spectrometry (GC-MS) to analyze breath VOCs. We used mice infected with Mucorales and uninfected mice as controls. We found the sesquiterpene secondary metabolites cedrene, selina-5,11-diene, and cedranoxide, 8, 14- to be distinctive in *Rhizopus microsporus* infection, β-isocomene, epicubebol, and γ-patchoulene to be distinctive in *Rhizopus arrhizus* var. *arrhizus* infection, and α-guaiene and alloaromadendrene to be distinctive in *Rhizopus arrhizus* var. *delemar* infection [86]. These unique breath-volatile metabolite profiles differentiated mice infected with these three species and also distinguished these mice from those infected with *A. fumigatus* and from healthy control mice. We also found that five patients with proven *Rhizopus microsporus* infection (all with pulmonary involvement, one who also had ROCM and two of whom also had systemic dissemination) had a profile of cedrene and 8, 14-cedranoxide (both found in the murine in vivo model), and 1H-Indene, 2,3,3a,4-tetrahydro-3,3a,6-trimethyl-1-(1-methylethyl)- in their breath. In patients who survived long enough to provide follow-up breath samples, this signature gradually dissipated in the patients who ultimately survived their mucormycosis and did not dissipate or increased in those who passed away from the dissemination of their disease. This approach has the potential for the noninvasive diagnosis of mucormycosis and for monitoring the in vivo response to antifungal therapy [86].

## 7. Conclusions

In conclusion, the difficulty of accurately diagnosing Mucorales infections persists. Initial workup of patients with suspected mucormycosis will almost always include conventional imaging studies which may reveal findings suggestive of these opportunistic fungi, but samples for fungal cultures should also be obtained whenever feasible. The decision of whether to pursue invasive procedures to collect BAL fluid samples or tissue biopsies will depend on the patient’s clinical syndrome and disease progression. Patients with suspected rhino-orbito-cerebral mucormycosis (ROCM) should undergo surgical debridement, and tissue samples must be sent for cultures and histopathology, including intraoperative frozen sections. Obtaining a biopsy from a patient with suspected pulmonary mucormycosis may prove to be more complex, and a considerable proportion of these patients are initiated on broad-spectrum antifungals without histopathology guidance. Species-level identification should be pursued whenever possible, particularly if isavuconazole or posaconazole are being considered as therapeutics instead of empiric broad-spectrum therapy with amphotericin B. Early administration of antifungals is, indeed, a critical factor impacting patient outcomes, and novel diagnostics could bridge the time gap between clinical suspicion and initiation of therapy. Faster and minimally invasive serology testing, molecular biology assays, and breath-based metabolomics show promise, though standardization and validation remain a barrier for the time being.
